# Do tumours located in basal segments have better survival than superior segments in lung cancer?

**DOI:** 10.1016/j.amsu.2022.104798

**Published:** 2022-11-01

**Authors:** Tianyi Lu, Jian Zhou, Mingying Lin, Jiandong Mei

**Affiliations:** a Department of Thoracic Surgery, West China Hospital, Sichuan University, Chengdu, China; b West China School of Medicine, Sichuan University, Chengdu, China

**Keywords:** Review, Lung cancer, Superior segment, Basal segment, Pulmonary surgery

## Abstract

A best evidence topic in thoracic surgery was written according to a structured protocol. The question addressed was in patients with lower lobe lung cancer undergone pulmonary resection, are the tumours located in superior segments superior to the tumours in basal segments in terms of survival? We concluded that there were no statistically significant differences in survival and recurrence between superior and basal segments for lung cancer patients, but overall survival and relapse-free survival were worse in superior segment for clinical stage I non-small cell lung cancer (NSCLC) in the right lower lobe, and remained unclear about other stages of lung cancer. In consideration of operation procedure, we speculate that the superior segments had a relatively worse survival in patients with early-stage NSCLC who underwent segmentectomy; likewise, in patients underwent at least lobectomy, survival of the superior segments was not better than that of the basal segments.

## Highlights

• No statistically significant differences in survival and recurrence exist between superior and basal segments for lung cancer patients.
• The superior segments had a relatively worse survival in patients with early-stage NSCLC who underwent segmentectomy.
• The survival rate of the superior segments was no better than that of the basal segments in patients underwent at least lobectomy.

## 1 Introduction

A best evidence topic was constructed according to a structured protocol. This is fully described in the International journal of surgery [1].

## 2 Clinical scenario

You are in the outpatient department of thoracic surgery. You notice that some patients have lower lobe lung cancer, and some of them have tumours in superior segments, some are in the basal segments. You are interested in the effect of different tumour locations on survival and decide to consult the literature yourself.

## 3 Three-part question

In [patients with lower lobe lung cancer] [undergone pulmonary resection], is [tumour located in superior segments superior to tumours in basal segments] in terms of [survival and recurrence]?

## 4 Search strategy

We performed a systematic search in Medline database using the PubMed interface from 1950 to September 2022, in Embase database using the OCID interface from 1974 to 2022 and in web of science with the terms: (“Lung Neoplasms"[Mesh]) and (“Pneumonectomy"[Mesh] or (Pneumonectom* [Title/Abstract]) Or lobectomy [Title/Abstract] or segmentectomy [Title/Abstract] or surgery [Title/Abstract] OR surgical [Title/Abstract] OR resect* [Title/Abstract]) and (segment [Title/Abstract] or (superior segment [Title/Abstract]) or (basal segment* [Title/Abstract]) or (basilar segment* [Title/Abstract]) or (segment 6 [Title/Abstract])) and (prognos* or Survival [Title/Abstract]). The results were limited to English articles and human studies.

## 5 Search outcome

**Fig. 1:**
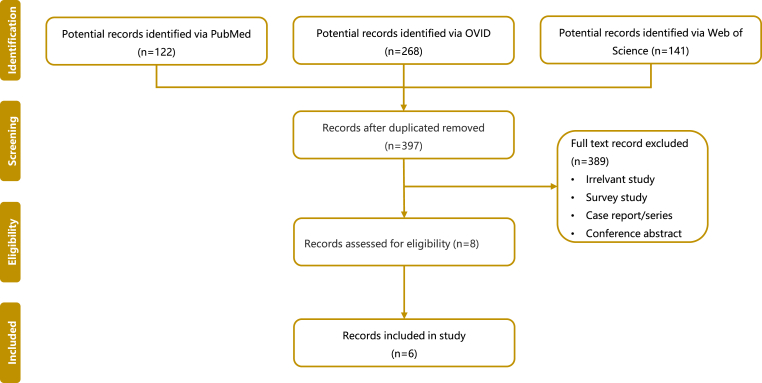
PRISMA flow chart.

**Five hundred and thirty-one** papers were found using the reported search. A total of 397 papers were found after removing duplicates. Among them, 389 papers were excluded according to the title and abstract, and the remaining 8 papers were screened and evaluated. From these, **six** retrospective cohort studies were identified that provided the best evidence to answer the question. The search strategy process is detailed in Fig. 1.

## 6 Results

See in Table 1.

**Table 1:** Best evidence papers.

Author, date of publication, journal and country	Study type (level of evidence)	Patient group	Outcomes	Key results	Additional comments
Yoshinori Handa et al., 2017, Ann Thorac Surg, Japan [2]	Retrospective cohort study (level III)	Sample size: n = 134 Median follow-up: 41.2 months Study period: 2007–2015 **Patients** clinical stage I (cN0M0) NSCLC - Superior group: n = 60 - Basal group: n = 74 **Treatment** Segmentectomy/lobectomy Superior group: 16/44 Basal group: 13/61	5-year OS	Total (78.1%) Superior group (62.6%) Basal group (89.9%) *P* = 0.0072	- A single centre study with a relatively large study population - More segmentectomy in superior group and tumour margins were not assessed
			Univariable analyses for OS (Superior group/Basal group)	HR 3.90 (95% CI 1.49–12.1) *P* = 0.005	
			Multivariable analyses for OS (Superior group/Basal group)	HR 3.33 (95% CI 1.22–13.5) *P* = 0.010	
			Incidence of recurrence	Superior group (26.7%) Basal group (10.8%) *P* = 0.0231	
			5-year RFS	Superior group (54.4%) Basal group (75.7%) *P* = 0.032	
			Univariable analyses for RFS (Superior group/Basal group)	HR 2.78 (95% CI 1.33–6.19) *P* = 0.006	
			Multivariable analyses for RFS (Superior group/Basal group)	HR 2.90 (95% CI 1.20–7.00) *P* = 0.008	
Jones, G. D. et al., 2021, Ann Thorac Surg, USA [3]	Retrospective cohort study (level III)	Sample size: n = 196 Study period: 2000–2018 Median follow-up: 4.9 years (IQR = 1.6–8.7) **Patients** cT1N0M0 NSCLC - Right basal group: n = 27 - Right superior group: n = 78 - Left basal group: n = 22 - Left superior group: n = 59 **Treatment** Segmentectomy	Multivariable analysis for DFS	Right superior group/Right basal group HR 2.89 (95%CI 1.18–7.08) *P* = 0.02 Left basal group/Right basal group HR 1.78 (95%CI 0.53–6.01) *P* = 0.35 Left superior group/Right basal group HR 2.09 (95%CI 0.80–5.43) *P* = 0.13	- A single centre study with a large sample size - The margin distance (cm) in the right basal group 2.5 (1.0–3.4) was greater than that in the right upper group 1.0 (0.5–2.5), which may cause more recurrence - A long-time span of 18 years may lead to heterogeneity in treatment modalities
			5-year DFS	Right superior group 57.6% (95% CI 45.7–72.7%) Right basal group 77.1% (95% CI 59.2–100%)	
			10-year DFS	Right superior group 17.9%, (95% CI 6.4–49.8%) Right basal group 57.9%, (95% CI 31.0–100%)	
			Multivariable analysis for OS	Right superior group/Right basal group HR 4.35 (95%CI 1.61–11.76), *P* = 0.004 Left basal group/Right basal group HR 3.59 (95%CI 0.99–13.01), *P* = 0.05 Left superior group/Right basal group HR 2.89 (95%CI 1.00–8.33), *P* = 0.05	
			5-year OS	Right superior group 66.3%, (95% CI 54.7–80.3%) Right basal group 79.5%, (95% CI 60.9–100%)	
			10-year OS	Right superior group 19.0%, (95% CI 6.9–52.5%) Right basal group 63.6%, (95% CI 38.1–100%)	
Tomizawa, K. et al., 2015, Thorac Cancer, Japan [5],	Retrospective cohort study (level III)	Sample size: n = 85 Study period: 1996–2012 Median follow-up: 38.8 months **Patients** pN2 lung cancer located in the right lower lobe - Superior group: n = 32 - Basal group: n = 53 **Treatment** Pneumonectomy/lobectomy Superior group:3/29 Basal group:1/52	3 - year DFS	Superior group (22.6%) Basal group (42.1%) *P* = 0.020	- Two centres study - Right lower lobe only - Small sample size - Mostly male (72.9%)
Watanabe, S. et al., 2008, Ann Thorac Surg, Japan [4],	Retrospective cohort study (level III)	Sample size: n = 139 Study period: 1981–2001 Median follow-up: **Patients** pN2 NSCLC - Superior group: n = 51 - Basal group: n = 88 **Treatment** Pneumonectomy/Lobectomy Superior group:23/28 Basal group:15/73	Overall 5-year survival	Total (27.9%) Superior group (19.9%) Basal group (32.9%) *P* = 0.1308	- Single centre study - No description of follow up procedure - More pneumonectomy in superior group (p < 0.01) may affect overall survival - When the tumour involved both the superior and the basal segments, the patient was placed in the superior segment group
Lin, Y. H. et al., 2019, Thorac Cancer, china Taiwan [6]	Retrospective cohort study (level III)	Sample size: n = 207 Study period: 2004–2013 Median follow-up: 33.9 months (Range = 3.2–110.8) **Patients** Lung adenocarcinoma - Superior group: n = 73 - Basal group: n = 134 **Treatment** lobectomy	5-year OS	Total 76.9%	- Largest number of study population in the evidence - Single centre study - Mostly early stage I patients - Basal group have more N2 (p = 0.025), larger tumour size (p = 0.052) may lead to worse prognosis
			Univariable analyses for OS (Basal group/Superior group)	HR 1.606 (95CI% 0.748–3.714), *P* = 0.212	
			5-year FFR	Total 56.5%	
			Univariable analyses for FFR (Basal group/Superior group)	HR 2.042 (95%CI 1.164–3.583) *P* = 0.013	
			Multivariable analyses for FFR (Basal group/Superior group)	HR 2.453 (95%CI 1.242–4.846) *P* = 0.010	
Nishio, W. et al., 2016, Ann Thorac Surg, Japan [7]	Retrospective cohort study (level III)	Sample size: n = 48 Study period: 1995–2009 Median follow-up: 107 months **Patients** cT1aN0M0 NSCLC - Superior group: n = 24 - Basal group: n = 24 **Treatment** Segmentectomy	Local recurrence	Superior group (4.2%) Basal group (20.8%)	- Small sample - Study population from another prospective cohort study may lead to selection bias - Baseline information of the two groups is unknown - Different number of segments were removed in basal group which may have influence on survival - *P* values were not provided
			Regional recurrence	Superior group (0%) Basal group (25.0%)	
			Local-regional recurrence	Superior group (4.2%) Basal group (37.5%)	
			5-year LRFS	Superior group (91.7%) Basal group (62.5%)	
			10-year LRFS	Superior group (69.5%) B (40.7%)	

NSCLC, non-small cell lung cancer; HR, hazard ratio; CI, confidence interval; IQR, interquartile range; OS, overall survival; RFS, recurrence-free survival; DFS, disease-free survival; FFR, freedom from recurrence; LRFS, local-regional recurrence-free survival.

## 7 Discussion

In total, data from a total of 1207 patients were analysed. Five-year overall survival rates were reported in three studies and ranged from 19.9% to 62.6% in superior segments and from 32.9 to 89.9% in basal segments. Some of the best available evidence obtained comes from a subset of patients in retrospective studies that were not designed to investigate this topic and are therefore of poor quality.

Yoshinori Handa et al. [2]conducted a study on 134 clinical stage I non-small cell lung cancer (NSCLC) patients with segmentectomy (n = 29) or lobectomy (n = 105) to confirm whether tumour location affected prognosis. This study supports a worse 5-year overall survival (OS) (*P* = 0.0072) and higher risk of recurrence (*P* = 0.0231) in the superior segment. Furthermore, among the patients who experienced postoperative recurrence, the superior segment group had more locoregional recurrences, compared with basal group. Subgroup analysis showed a similar trend in the patients with pathologically stage I lung cancer and the patients who undergone lobectomy. Poorer 5-year OS in superior segment was also found in patients with right side tumour (n = 90). Handa et al. [2] speculated that anatomic proximity of the superior segment to the hilar structures, and more aggressive biological behaviour might contribute to their results.

Jones et al. [3] retrospectively reviewed 416 cT1N0M0 NSCLC patients who received intentional segmentectomy. Patients were divided into seven groups based on tumour location, four of which were relevant to this topic: right basal segments (n = 27), right superior segment (n = 78), left basal segments (n = 22), left superior segment (n = 59). Right superior group was independently associated with worse disease-free survival (DFS) (vs right basal group, *P* = 0.02) and OS (vs. right basal group, *P* = 0.004) in multivariable analysis. DFS and OS of left basal group and right basal group were not statistically different. The authors did not differentiate bilateral basal and superior segments. It is worth noting that the baseline characteristics were not well balanced and the mean margin distance in right superior group were significantly smaller than that in right basal group (1.0 vs. 2.5 cm), which may contribute to worse DFS and OS in right superior group. In addition, the right superior group had a large proportion of later-stage tumours, lymphovascular invasion and aggressive lung adenocarcinoma. In addition, a long time-span of 18 years may lead to heterogeneity in treatment modalities. The author stated a lower survival and recurrence-free rates for tumours in the right superior segment, compared with the tumours in other segments in lower lobe. Thus, the patients with right superior segment tumours may warrant more extensive resection. These 2 studies consistently indicated an increased recurrence rate and a decreased survival rate in the superior group.

Watanabe et al. [4] compared the survival outcomes of 139 patients with pN2 NSCLC and lobectomy (n = 101) or pneumonectomy (n = 38) in relation to the location of lesion. They found no statistical difference in 5-year survival outcomes between superior and basal group (19.9% vs 32.9%, *P* = 0.1308). The authors suggest that the relatively worse survival in superior group may be explained by the fact that there were more pneumonectomies in superior group. Besides, when the tumour involved both the superior and basal segments, Watanabe and associates assigned the patient to the superior segment, as opposed to the segment with main tumour volume in other studies. This grouping criteria may place more patients with invasion of both lung segments into the superior segment group, skewing the superior segment results. The author suggested that for patients with pN2 NSCLC, survival may be poorer for those with superior segment tumours than that of those with basal segment tumours.

Tomizawa et al. [5] studied 263 patients with lung cancer located in the right lower lobe underwent lobectomy (n = 81) or pneumonectomy (n = 4). In pN2 patients (n = 85), a lower 3-year DFS was found in right superior group (n = 32) compared with that in right basal group (n = 53) (22.6% vs 42.1%, *P* = 0.020). This result was consistent with the previous conclusion that the prognosis is worse in the superior segment. Nevertheless, among all patients (n = 263), pN0 (n = 153) and pN1 (n = 25) tumour patients, there was no significant difference in DFS between the right superior group and right basal group.

Lin et al. [6] reviewed 207 patients with lung adenocarcinoma who underwent lobectomy. Their results also suggested that tumour location was not an independent prognostic factor of OS (*P* = 0.212). Notably, they also found that basal group was associated with a lower probability of freedom from recurrence (basal vs. superior, HR = 2.453 (95%CI 1.242–4.846), *P* = 0.01). However, we cannot ignore the fact that basal group had larger tumours on average and more patients with N2 lymph node metastasis (*P* = 0.025), as well as the influence of these two factors on recurrence.

Nishio et al. [7] studied 237 patients with cT1aN0M0 NSCLC and performed a segmentally survival analysis on the patients undergoing segmentectomy, among which 48 patients with superior (n = 24) and basal (n = 24) segments tumours were matched with our topic. It was demonstrated that tumours in superior segments have a lower local and regional recurrence probability were than basal segments. However, *P* values were not provided, and the sample size was small. Therefore, the quality of this evidence needs to be considered.

Taking the operation types as one of the factors, two of six studies focus on the outcomes of patients with cT1N0M0 NSCLC who underwent segmentectomy and reached inconsistent conclusions. Given the study design and sample size, we tend to assume that the superior segments had a relatively worse survival in patients with early-stage NSCLC who underwent segmentectomy. In the other four studies, patients were treated with at least lobectomy. Although these studies focused on different stages and had conflicting primary results, based on the available evidence, we take the attitude that the survival of the superior segments was no better than that of the basal segments.

## 8 Clinical bottom line

Three of six studies verified no significant difference in overall survival between right or bilateral superior and basal segments for lung cancer. Two studies suggested a poorer survival and recurrence in clinical stage I NSCLC of right superior segment, another suggested a worse survival of superior segment for pN2 right-sided lung cancer. Two studies showed that the basal segments had a higher probability of local and regional recurrence while the results of the other three studies supported a higher likelihood of recurrence of superior segment. Three of six studies supported inferior survival of superior segments compared to the basal segments for specific population. However, the evidence was not sufficient enough due to the small sample size and greatly varied tumour size and staging.

In summary, we hold the opinion there were no statistically significant differences in survival and recurrence between superior and basal segments for lung cancer patients, but overall survival and relapse-free survival were worse in superior segment for clinical stage I NSCLC, and remained unclear about other stages of lung cancer. In consideration of operation procedure, we speculate that the superior segments had a relatively worse survival in patients with early-stage NSCLC who underwent segmentectomy; likewise, in patients underwent at least lobectomy, survival of the superior segments were no better than that of the basal segments.

## Provenance and peer review

Not commissioned, externally peer reviewed.

## Ethical approval

Ethical approval was not required.

## Source funding for your research

This study was supported by the 1.3.5 Project for Disciplines of Excellence, 10.13039/501100013365West China Hospital, Sichuan University (ZYJC18009 to Dr. Jiandong Mei).

## Author contribution

Tianyi Lu: conducted the literature search and writing of the paper.

Jian Zhou: conducted the data collection, analysis and writing of the paper.

Mingying Lin: assisted in the literature search and writing of the paper.

Jiandong Mei: assisted in the editing and writing of the paper.

## Registration of research studies

Name of the registry:

Unique Identifying number or registration ID:

Hyperlink to your specific registration (must be publicly accessible and will be checked):

## Guarantor

Jiandong Mei.

## Consent

Consent was not required.

## Declaration of competing interest

No potential conflict of interest was reported by the authors.
